# Immunomodulatory activity of a novel polysaccharide from *Lonicera japonica* in immunosuppressed mice induced by cyclophosphamide

**DOI:** 10.1371/journal.pone.0204152

**Published:** 2018-10-08

**Authors:** Xiaonan Zhou, Qun Dong, Xianzhao Kan, Lihong Peng, Xingyu Xu, Yun Fang, Jialiang Yang

**Affiliations:** 1 Key Laboratory of Polysaccharide Drug Engineering of Anhui, Wannan Medical College, Wuhu, Anhui, P. R. China; 2 College of Life Sciences, Anhui Normal University, Wuhu, P. R. China; 3 College of Information Engineering, Changsha Medical University, Changsha, Hunan, P. R. China; 4 College of Life Sciences, Zhejiang Sci-Tech University, Hangzhou, P. R. China; 5 Department of Mathematics, Shanghai Normal University, Shanghai, P. R. China; 6 Icahn Institute for Genomics and Multiscale Biology, Icahn School of Medicine at Mount Sinai, New York, NY, United States of America; University of Manchester, UNITED KINGDOM

## Abstract

*Lonicera japonica* is a typical Chinese herbal medicine. We previously reported a method to isolate polysaccharides from *Lonicera japonica* (LJP). In this study, we first performed a qualitative analysis of LJP using the Fourier Transform Infrared Spectrometer (FT-IR) and explored the monosaccharide composition of LJP using the pre-column derivatization high performance liquid chromatography (HPLC) method. We then investigated the immunomodulatory function of LJP in cyclophosphamide (CTX)-induced immunosuppressed mouse models. The results showed that LJP had the characteristic absorption of typical polysaccharides consisting of 6 types of monosaccharides. In addition, LJP can increase significantly the organ index, splenic lymphocyte proliferation, macrophage phagocytosis, and natural killer (NK) cell activity in CTX-treated mice. LJP could also restore the levels of serum cytokines interleukin (IL-2), tumor necrosis factor (TNF-α) and Interferon-γ (IFN-γ) in the CTX-treated mice. Finally, the results on measuring the T-lymphocytes subsets of spleen also confirmed LJP-induced immunomodulatory activity in immunosuppressed mice from another perspective. Therefore, LJP could be used as a potential immunomodulatory agent.

## 1. Introduction

Polysaccharides are a kind of natural polymer linked by aldose or ketose through glycosidic bonds. As one of the basic substances to maintain the normal functioning of life, polysaccharides are important biological macromolecules *in-vivo* [1]. In addition, polysaccharides have medicinal functions including immunomodulatory function, anti-bacterial, anti-virus, and anti-tumor [2,3]. For example, *polygonatum sibiricum* polysaccharide can enhance T cell and B cell proliferation responses and restore the levels of IL-2, TNF-α, IL-8 and IL-10 in the serum of the Cyclophosphamide (CTX)-treated mice [4]. *Lycium ruthenicum Murr*. polysaccharide can restore the levels of interleukin-2 (IL-2), IL-6 and tumor necrosis factor-α (TNF-α) in the serum of CTX-treated mice [5]. *Hyriopsis cumingii* polysaccharides could strengthen peritoneal macrophage expressing MR-1 and NF-κB in a dose-dependent manner [6]. In fact, several polysaccharides, such as *Ginseng* polysaccharides [7], *Cheonggukjang* polysaccharides, *Trametes orientalis* polysaccharide [8], *Ganoderma atrum* polysaccharide [9], have already been used clinically to enhance the body's immune function.

As one of the commonly used Chinese herbal medicines [10], *Lonicera japonica* has anti-bacterial, anti-oxidation, anti-allergy and immunoregulation effects. It can be used to treat respiratory infections, bacillary dysentery, acute urinary tract infections, and high blood pressure [11]. *Lonicera japonica* polysaccharide (LJP) is one of the main active ingredients of *Lonicera japonica*. However, the functions of this polysaccharide are still not fully understood especially on immune systems. Hence, it is necessary to study the role of LJP on the immune function of immunosuppressed mice induced by CTX in order to better develop this Chinese herbal medicine plant.

In a previous study, we isolated and characterized polysaccharides from *Lonicera Japonica*. The objective of this study is to conduct a qualitative analysis of LJP, explore its monosaccharide composition, and investigate its immunomodulatory function in CTX-induced immunosuppressed mice models. The ameliorative effects of LJP will be estimated by organ index, splenic lymphocyte proliferation, macrophage phagocytosis, natural killer (NK) cell activity, serum cytokines interleukin (IL-2), tumor necrosis factor (TNF-α) and Interferon-γ (IFN-γ) content, and T-lymphocytes subsets of spleen in CTX-treated mice.

## 2. Materials and methods

### 2.1 Ethics statement

All experimental animal procedures were carried out in strict accordance with the National Institutes of Health Guide for the Use of Laboratory Animals. Procedures involving animals and their care were approved by the Animal Ethics Committee of the Yijishan Hospital of Wannan Medical College (SCXK 2017–0006).

### 2.2 Materials and chemicals

All experiments were carried out on male mice aged from 8 to 12 weeks. The animals were housed under standard conditions with a 12 h light/dark cycle and adequate water and food. Male Balb/c mice (20.8 ± 2.3 g) were obtained from the Experimental Animal Center of Wannan Medical College (Wuhu, China). *Lonicera Japonica* was purchased from Laobaixing chemist's shop in Wuhu, China. Standard monosaccharides, including D-glucose, D-fucose, D-mannose, D-rhamnose, D-galactose, D-xylose and D-arabinose were bought from Sigma (St. Louis, MO, USA). Rosewell Park Memorial Institute (RPMI) 1640 medium and fetal bovine serum (FBS) were purchased from GIBCO BRL (Grand Island, NY, USA). The concanavalin A (ConA), dimethyl sulfoxide (DMSO), lipopolysaccharide (LPS), 3-(4, 5-dimethylthiazol-2-yl)-2, 5-diphenyltetrazolium bromide (MTT) were purchased from Sigma–Aldrich (St. Louis, MO, USA). YAC-1 cells were purchased from purchased from Cell Bank of Chinese Academy of Science (Shanghai, China). FITC-CD3^+^, PE-CD4^+^ and APC-CD8^+^ antibodies were purchased from BioLegend, Inc. (San Diego, CA). CTX and Enzyme linked immunosorbent assay (ELISA) kits for IL-2, NF-α and IFN-γ were obtained from BOMEI Biotechnology Co., Ltd (Hefei, China).

### 2.3 Preparation of the LJP

The dried *Lonicera Japonica* powder (100 g) was added into 2 L of distilled water and 1.5 g of cellulose. The mixture was placed at 45°C for 50 min to extract the polysaccharides. The water extract was then placed in 90°C water bath for 10 min to inactivate the enzyme. After inactivation of the enzyme, the water extract was cooled to room temperature and centrifuged for 10 min at 2655 ×g using a refrigerated centrifuge. The supernatant was concentrated to 500 mL and 2 L 95% (w/w) ethanol solution was added into it. The supernatant was placed at 4°C for 12 h and then centrifuged for 10 min at 2655 ×g using a refrigerated centrifuge. 10 mL distilled water, 2 mL of chloroform and 0.5 mL of n-butanol were added into the precipitate to remove the protein. Finally, the solution was lyophilized to gain LJP.

### 2.4 FT-IR measurement analysis

The LJP was measured using a fourier transform infrared spectrometer (IS10, Thermo Nicolet Corporation, Madison, USA). 2 mg of the freeze-dried LJP sample and 100 mg of potassium bromide were added into a mortar and ground into a uniform powder. The mix powder was pressed into thin slices for the full-band infrared spectrometer scan (4000–400 cm^-1^). The scanning times were set to be 64. Data were processed using the Omnic software (Nicolet, USA) and the Origin 8.0 software (Origin Lab Corp., Northampton, MA, USA).

### 2.5 Monosaccharide composition analysis

The monosaccharide composition of LJP was analyzed using the pre-column derivatization HPLC method [12]. 10 mg LJP was hydrolyzed to prepare monosaccharides by using 2 mol/L trifluoroacetic acid (TFA, 2.0 mL) at 121°C for 1 h in a sealed ampule. After hydrolysis, TFA was evicted by vacuum concentrator. Then, the dried sample was dissolved in distilled water (2.0 mL). The aqueous phase was diluted 50 times for the HPLC analysis. Monosaccharide compositions of polysaccharides were performed on a Dionex Ion Chromatograph 2010i system (Dionex, USA), which contains advanced gradient pump and an eluent degas module. The mobile phase was composed of phosphate buffer (50 mmol/L, pH 6.8) and acetonitrile with the volume ratio of 78:22. The flow-rate was 1.0 mL/min. Standard monosaccharides were subjected to the same conditions described above as a reference. According to the time of each chromatographic peak in the chromatogram, the monosaccharide composition of a sample can be determined. The molar ratio of various monosaccharides can be calculated from the peak areas of the respective chromatographic peaks.

### 2.6 Mice treatment and experimental design

After 7 days of acclimatization, mice were randomly divided into the control group, the CTX model group, the LJP low (LJP-L), medium (LJP-M) and high (LJP-H) dose groups (12 in each group). Except for the control group, mice in other four groups were subjected to immunosuppression by intraperitoneal injection of CTX (70 mg/kg/d) from day 1 to 4. From day 5 to 14, the mice in the LJP low, medium and high dose groups were given 1 mL of LJP aqueous solution at different dosages of 50 mg/kg, 100 mg/kg and 150 mg/kg body weight daily by gavage. The mice in the control group and the CTX model group were given 1mL distilled water daily by gavage. Twenty-four hours after the last gavage, the mice were then weighed and sacrificed for further measurements.

### 2.7 Measurement of spleen and thymus indices

After the mice were sacrificed, their spleen and thymus were collected and weighted immediately to calculate the spleen and thymus indices. The spleen and thymus indices were calculated according to the formula: Spleen or thymus indices (mg/g) = weight of spleen or thymus (mg)/weight of mouse.

### 2.8 Preparation of mouse spleen lymphocyte suspension

Mouse spleen was isolated under aseptic conditions. After fat and connective tissue were removed, the spleen was added with physiological saline and ground in a mortar. The mixture was then filtered. The filtrate was centrifuged at 238 ×g for 10 min. The precipitate was added with red blood cells lysis solution and placed on ice for 10 min. The mixture was centrifuged at 238 ×g for 5 min. The cells were washed twice with phosphate buffer saline and then added into 5 mL RPMI-1640 medium. Finally, the cells were adjusted to the concentration of 2 × 10^5^/mL.

### 2.9 Measurement of splenic lymphocyte proliferation

Mice spleen lymphocyte suspension (2×10^5^/mL) was used for the measurement of splenic lymphocyte proliferation. Mice spleen cell suspension was seeded in 96-well plates and then ConA (5 μg/mL) or LPS (8 μg/mL) were added into each well. The plates were placed in 37°C incubator for 4 h, and then 20 μL (5 mg/mL) MTT solution was added into each well. After incubating for 4 h, the supernatant was abandoned. Each well was added with DMSO (150 μL) and shaken for 10 min. The OD values at 490 nm were measured with a microplate reader. The stimulation index (SI) was calculated as SI = Stimulus OD value/Control OD value.

### 2.10 Measurement of macrophage phagocytosis

Twenty-four hours after the last gavage, the mice were injected intraperitoneally with 0.5 mL of 3% (v/v) chicken red blood cells (CRBCs) suspensions. Sixty min later, the mice were sacrificed by dislocation and intraperitoneal injected with 2 mL physiological saline. Peritoneal exudates (0.5 mL) were spread on glass slides and incubated at 37°C for 30 min. After that, the glass slides were rinsed with physiological saline to remove the non-phagocytosed CRBCs. The macrophages were fixed with 1:1 acetone-methanol solution and stained with 4% (v/v) Giemsa for 15 min. After drying, the macrophages were observed and counted with microscope. The phagocytic rate and phagocytic index were calculated according to the following formula: The phagocytic rate (%) = (Number of macrophages phagocytized CRBCs/Total number of macrophages) × 100%. The phagocytic index = Number of CRBCs phagocytized by macrophages /Total number of macrophages.

### 2.11 Measurement of nature killer cell (NK) cytotoxicity

Mouse spleen lymphocyte suspension (2×10^5^/mL) was used as the effector cells. YAC-1 (4×10^4^/mL) cells were used as the target cells. The effector cells and target cells were seeded into 96-well plates at a ratio of effector to target cells of 50:1. The cells were then incubated at 37°C for 5 h in a humidified incubator containing 5% CO_2_. After incubating for 5 h, 20 μL of MTT solution (5 mg/mL) was added to the incubator and the culture was continuing for 4 h. The supernatants (100 μL of each) were added to another 96-well plates and 50 μL of DMSO was added to each well. The 96-well plates were shook for 15 min. The optical density (OD) at 490 nm was detected by a microplate reader. The killing activity of NK = [Target cell—(Effect-target cell OD value—Effects OD value)/Target cell OD value] x 100%.

### 2.12 Measurement of serum cytokines IL-2, TNF-α, IFN-γ content

The whole blood of mice was collected and centrifuged at 955 ×g for 10 min to draw the upper serum. The serum cytokine levels (IL-2, TNF-α, IFN-γ) were detected by the ELISA kits according to the instruction from the manufacturer.

### 2.13 Measurement of T-lymphocytes subsets of spleen

Mouse spleen lymphocyte suspension (2×10^5^/mL, 100 μL) was added to the flow EP tube. After that, FITC-CD3^+^, PE-CD4^+^, and APC-CD8^+^ antibodies (each 4 μL) were added. The tube was blended and placed at 4°C dark place for 60 mins. Cells were then washed twice with PBS and centrifuged at 238 ×g for 5 min. 300 μL PBS was then added to the precipitate, which was determined by flow cytometry (BD FACS Aria II, NJ, USA). The percentage of each labeled cell in lymphocytes was calculated using the FlowJo software.

### 2.14 Statistical analysis

The software SPSS 20.0 (IBM, Chicago, IL, USA) was used to perform the analysis of variance (ANOVA). One-way analysis of variance was used to determine the significant difference between mean values. A 95% confidence level (*P* < 0.05) was considered to be statistically significant.

## 3. Results

### 3.1 The FTIR analysis of LJP

**Fig 1:**
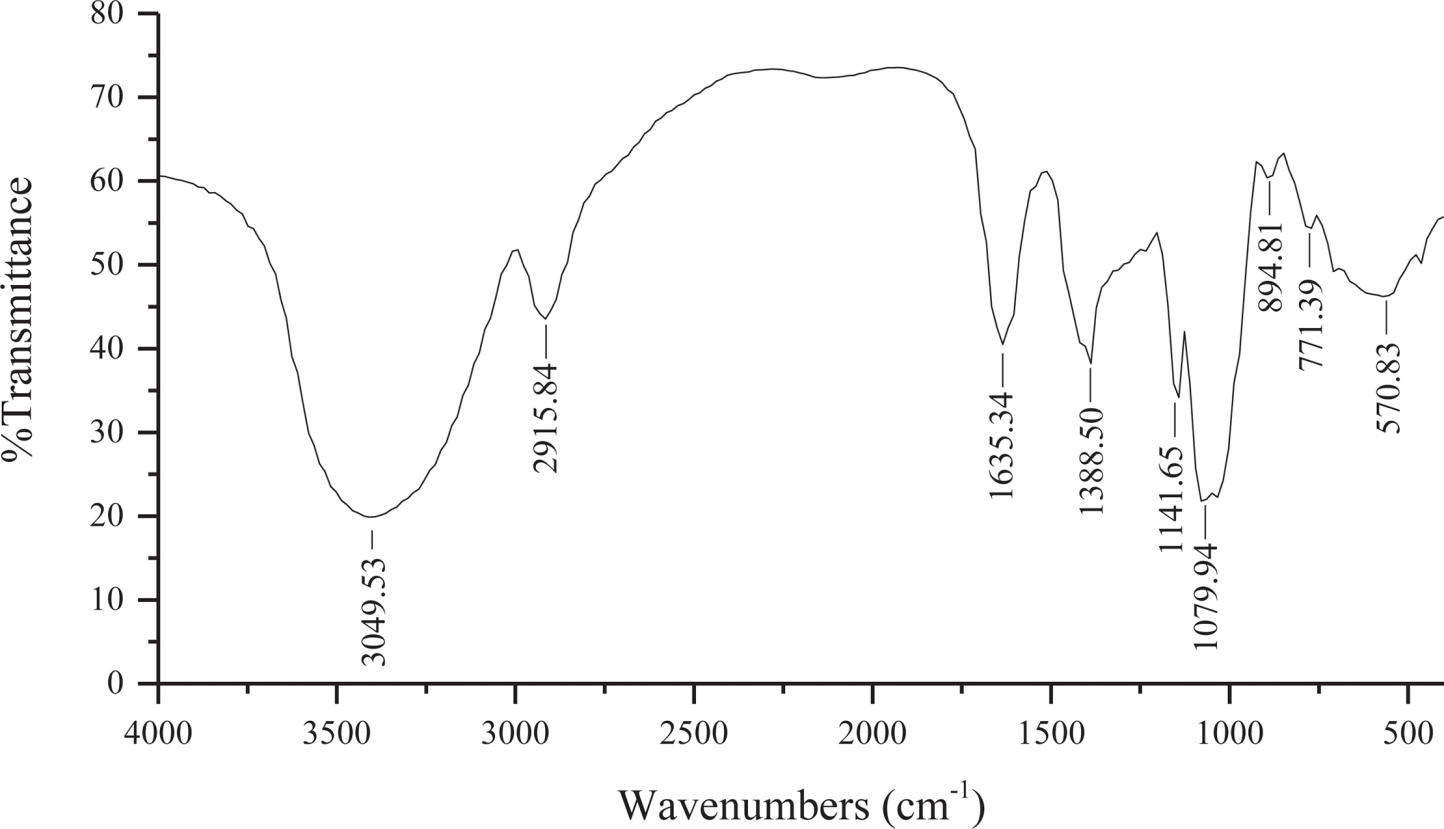
FT-IR spectrum of LJP in the range of 4000–400 cm^−1^.

Fig 1 shows the FT-IR spectrum of LJP in the range of 4000–400 cm^−1^. LJP has a broad and strong absorption peak at around 3400 cm^-1^, which is caused by O-H bond vibration in the sugar compound. At the same time, LJP also has a weaker absorption peak at near 2900 cm^-1^, which is caused by vibration of C-H bonds in sugar compounds. These two absorption peaks are characteristic absorption peaks of sugar compounds [4]. The absorption peak at 1635 cm^-1^ is a characteristic absorption peak of the crystalline water of sugar compounds. The peak at 1388 cm^−1^ was also the characteristic absorptions of C-H bonds. The stretching peaks at 1141 and 1079 cm^−1^ were due to the presence of C-O glycosidic bonds. The absorption peaks near 894 cm^−1^ represent the β-configuration and α-configuration. In addition, the small band at around 771 cm^−1^ was attributed to the C-O-C symmetry vibration peak of D-glucopyranosyl ring [13,14]. These results suggest that LJP had the characteristic absorption of typical polysaccharides.

### 3.2 Monosaccharide composition of LJP

Monosaccharides are the basis of the chemical structure of polysaccharides, which may contribute into the biological activity of polysaccharides. Thus, it is very important to analyze the monosaccharide composition of LJP. Table 1 shows the result of monosaccharide composition of LJP. The results implied the dominance of glucose (Glu), galactose (Gal), mannose (Man), rhamnose (Rha), xylose (Xyl) and arabinose (Ara) in LJP.

**Table 1:** Monosaccharide compositions of LJP.

	Mol%					
	Glu	Gal	Man	Rha	Xyl	Ara
LJP	58.62	9.17	2.89	5.33	3.26	20.73

### 3.3 Effect of LJP on spleen and thymus indices

**Fig 2:**
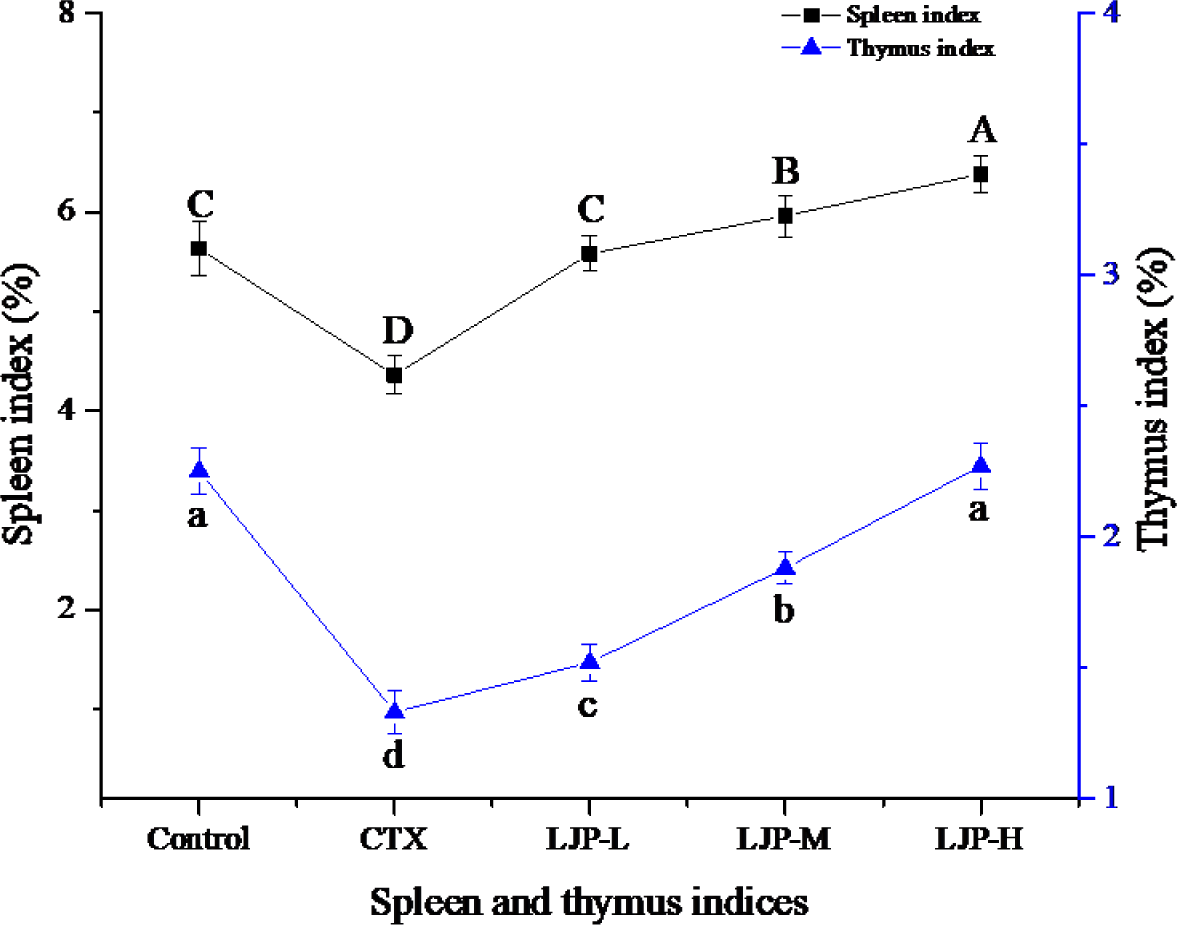
Effect of JPL on spleen and thymus indices of immunosuppression mice induced by CTX. Different letters within the same index express the significant differences (*P* < 0.05).

Fig 2 shows the effect of LJP on spleen and thymus indices of immunosuppression mice induced by CTX. Compared to the control samples, the spleen and thymus indices of the CTX-treated group were reduced significantly (with *P* < 0.05). As the dosages of LJP increased, the spleen and thymus indices of mice also rose significantly (*P* < 0.05). The results suggest that LJP increases the weight of immune organs in mice and can enhance the immune function of mice.

### 3.4 Effect of LJP on the splenic lymphocyte proliferation

**Fig 3:**
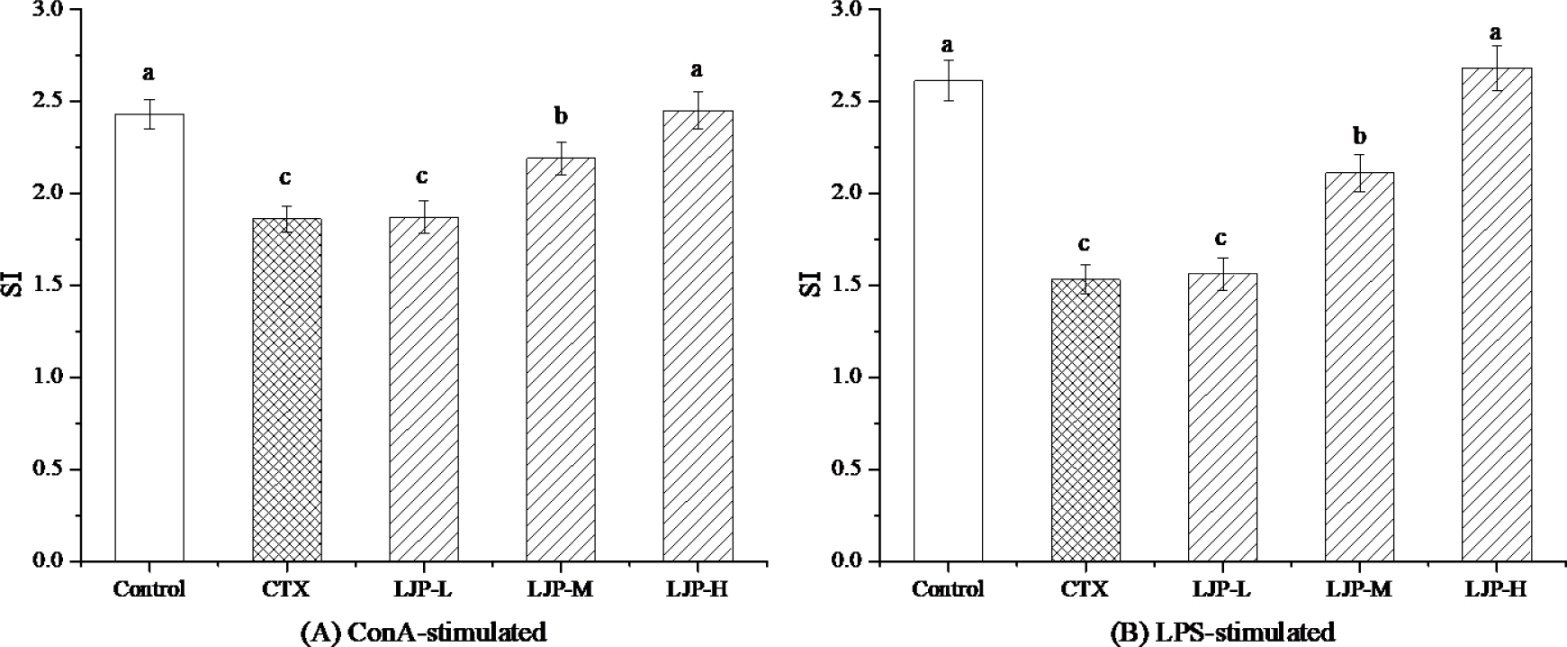
Effect of LJP on the splenic lymphocyte proliferation in immunosuppression mice induced by CTX. Different letters within the same index express the significant differences (*P* < 0.05).

Fig 3 shows the effect of LJP on the splenic lymphocyte proliferation in immunosuppression mice. Compared to the control group, the splenic lymphocyte proliferation in the CTX group was significantly reduced (*P* < 0.05). The LJP-M and LJP-H can improve the splenic lymphocyte proliferation of immunosuppressive mice induced by CTX (*P* < 0.05), while LJP-L activity of immunosuppressed mice was not increased significantly (*P* > 0.05).

### 3.5 Effect of LJP on macrophage phagocytosis

As can be seen from Table 2, the macrophages phagocytic rate and phagocytic index of CTX group were significantly decreased (*P* < 0.05). Compared to the CTX group, the phagocytic rates of the LJP-L, LJP-M and LJP-H groups were significantly increased (*P* < 0.05), especially in the LJP-H group, where the phagocytosis rate was increased by 30.38%. Compared to the CTX group, the phagocytic indices of the LJP-L and LJP-M groups showed no significant difference, but the phagocytic index of the LJP-H group was significantly higher than that of the CTX group (*P* < 0.05). In summary, the addition of LJP improved the macrophages phagocytic rate and phagocytic index of immunosuppression mice induced CTX.

**Table 2:** Effect of LJP on macrophage phagocytosis in immunosuppression mice induced by CTX.

Groups	Numbers of mice	Phagocytosis rate (100%)	Phagocytosis index
Control	12	48.61±1.35^a^	0.59±0.02^a^
CTX	12	31.67±1.23^d^	0.43±0.03^c^
LJP-L	12	35.37±1.07^c^	0.47±0.03^c^
LJP-M	12	36.82±0.99^c^	0.48±0.05^c^
LJP-H	12	41.29±1.54^b^	0.52±0.03^b^

Results are mean ± standard deviation. Different lowercase letters in the same column within the same sample express the significant differences (*P* < 0.05).

### 3.6 Effect of LJP on NK cytotoxicity

**Fig 4:**
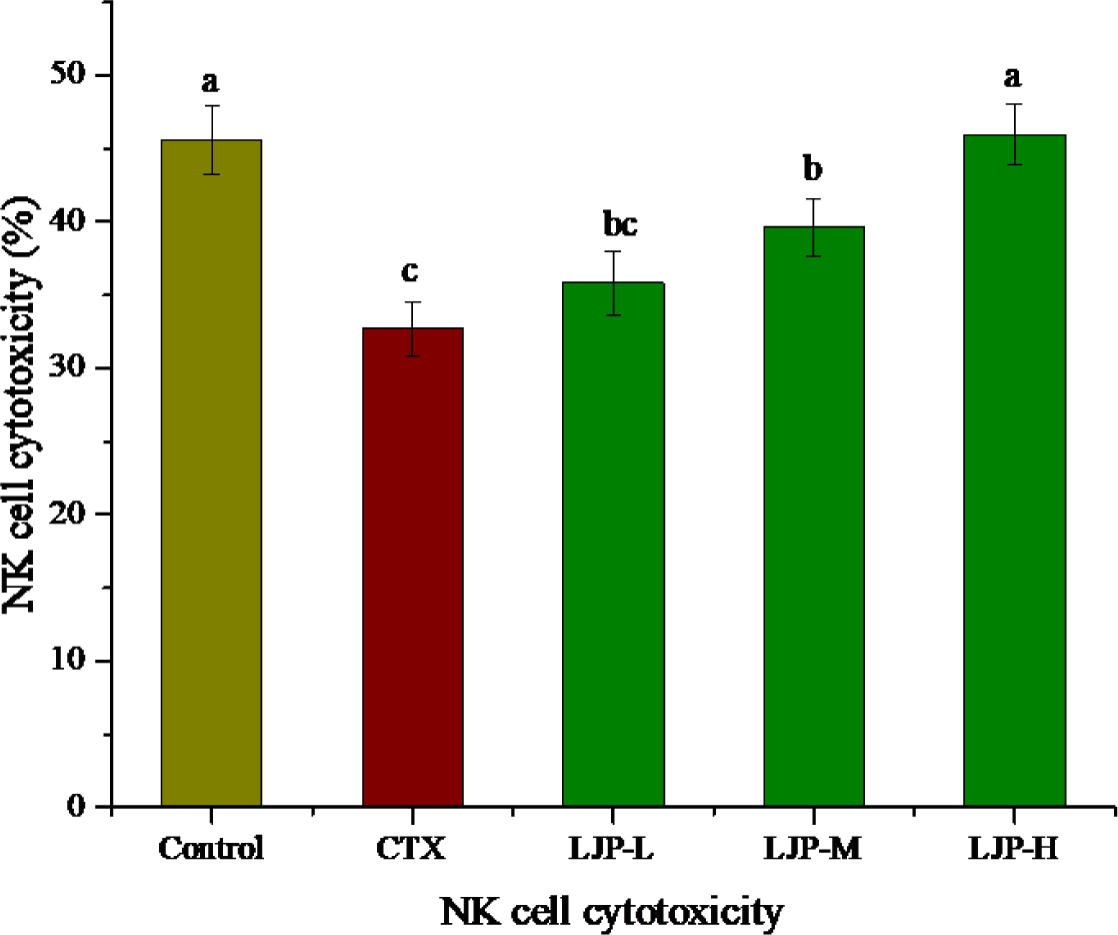
Effect of LJP on the NK cytotoxicity in immunosuppression mice induced by CTX. Different letters within the same index express the significant differences (*P* < 0.05).

Fig 4 shows the effect of LJP on NK cytotoxicity. The NK cytotoxicity of the CTX group was significantly decreased (*P* < 0.05), indicating that the immunosuppressed model of mice induced by CTX was successfully established. Compared to the CTX group, the NK cytotoxicity of the LJP-L group showed no significant difference, but the NK cytotoxicity of the LJP-M and LJP-H groups was significantly higher than that of the CTX group (*P* < 0.05). In particular, the NK cytotoxicity of the LJP-H group reached the same level as the control group.

### 3.7 Effect of LJP on serum IL-2, TNF-α, and IFN-γ concentration

**Fig 5:**
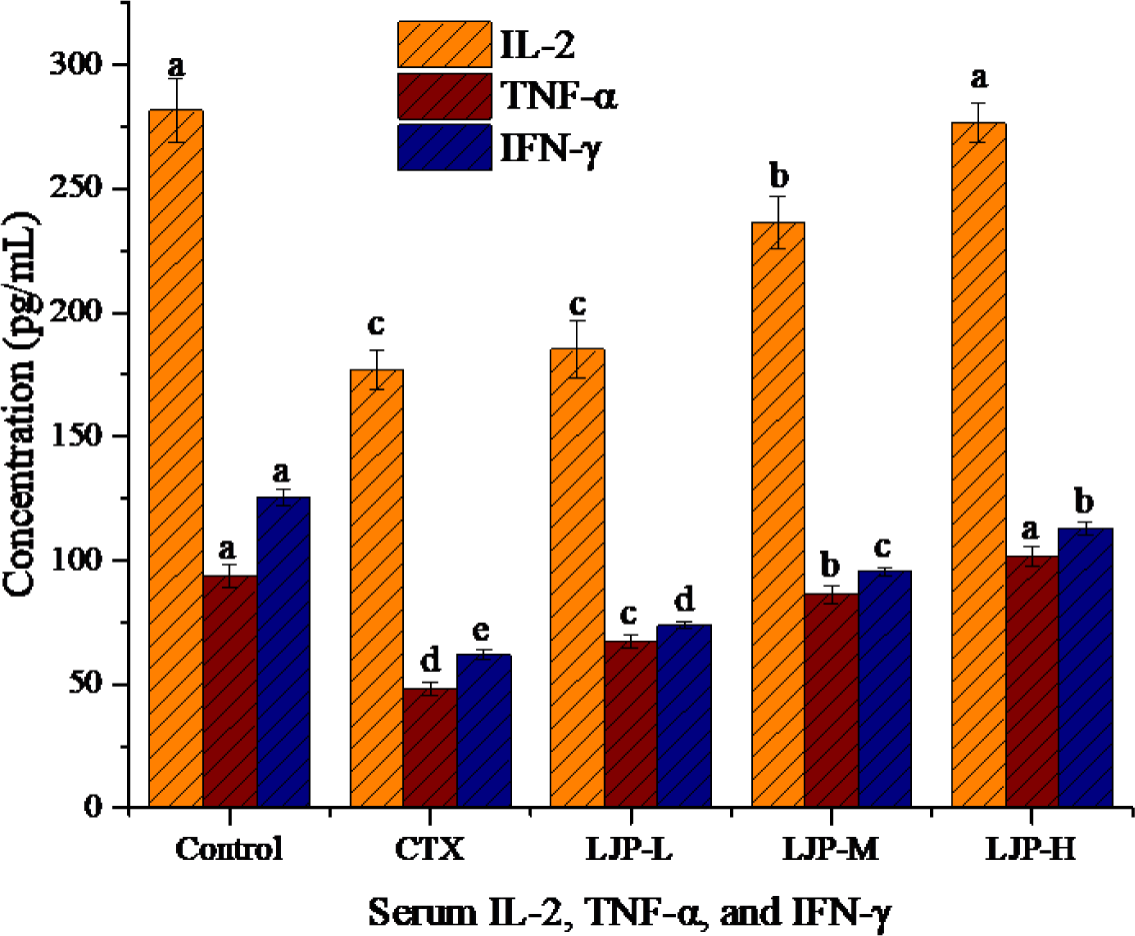
Effect of LJP on the serum IL-2, TNF-α, and IFN-γ concentration in immunosuppression mice induced by CTX. Different letters within the same index express the significant differences (*P* < 0.05).

As can be seen from Fig 5, the concentration of the serum IL-2, TNF-α, and IFN-γ in the CTX-treated mice decreased significantly (*P* < 0.05). Compared to the CTX group, the concentration of IL-2 in the LJP-L group showed no significant difference, but the concentrations of IL-2 in the LJP-M and LJP-H groups were significantly higher than that of the CTX group (*P* < 0.05). The concentrations of TNF-α and IFN-γ in mice increased with the dose concentration of LJP. The concentration of TNF-α in the LJP-H group had no significant difference with that of the control group.

### 3.8 Effect of LJP on T-lymphocytes subsets of spleen

**Fig 6:**
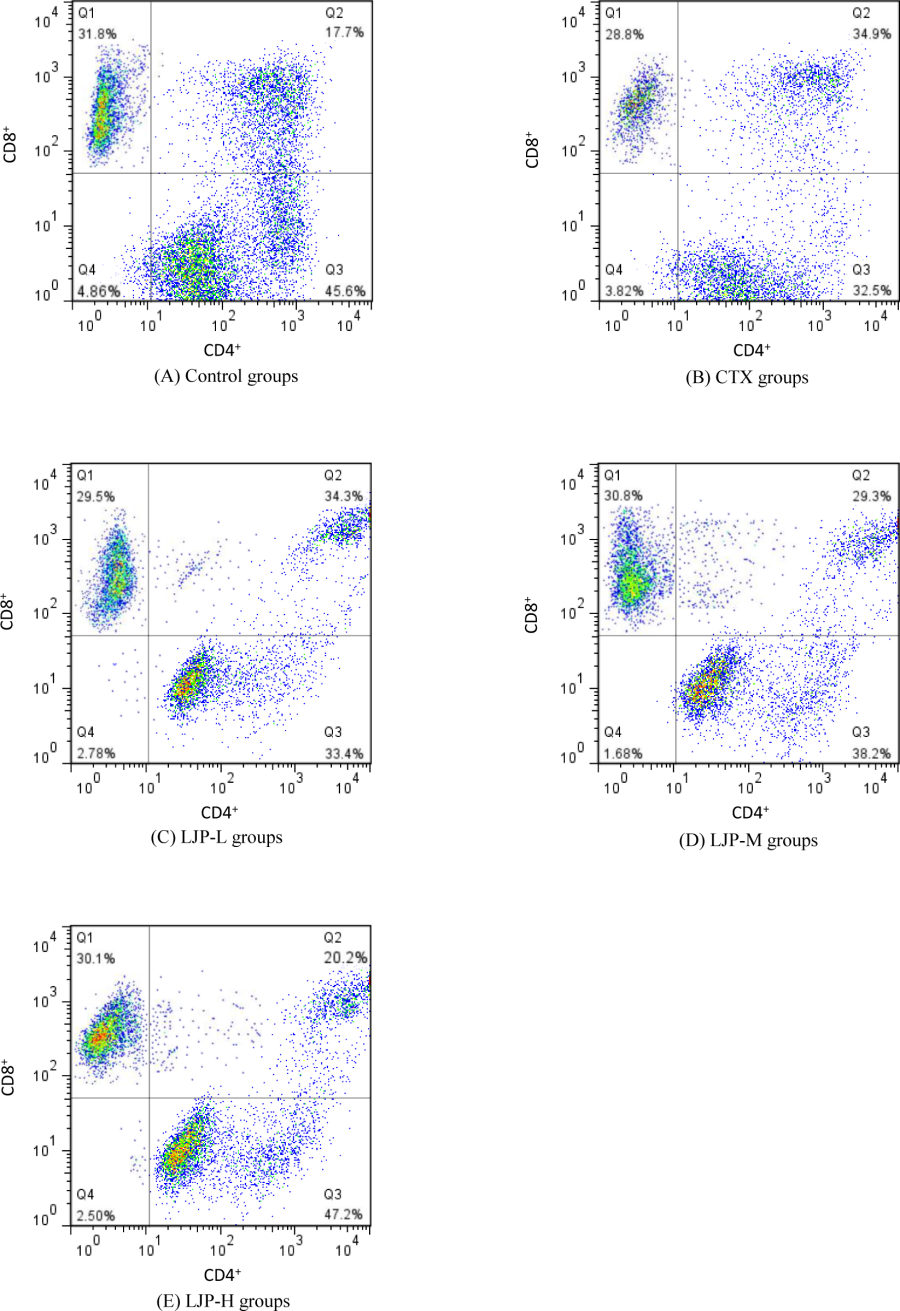
Effect of LJP on the T-lymphocytes subsets of spleen in immunosuppression mice induced by CTX. Different letters within the same index express the significant differences (*P* < 0.05).

The experimental results were shown in Table 3 and Fig 6. Compared to the control group, the percentages of CD4^+^ and CD8^+^ T cells in the CTX mice decreased significantly (*P* < 0.05). Mice cellular immune function was inhibited, indicating that immunosuppressive mice model was successfully constructed. Compared to the CTX group, the percentages of CD4^+^ and CD8^+^ T cells and the ratio of CD4^+^/CD8^+^ in the LJP group increased significantly (*P* < 0.05). The ratio of CD4^+^/CD8^+^ in the LJP-H group had even reached the same level as that of control group. In summary, LJP can increase the level of CD4^+^/CD8^+^ T cell, which directly enhances the cellular immune function in the CTX immunosuppressed mice.

**Table 3:** Effect of LJP on the T-lymphocytes subsets of spleen in immunosuppression mice induced by CTX.

Groups	CD3^+^ (%)	CD4^+^ (%)	CD8^+^ (%)	CD4^+^/ CD8^+^
Control	75.36±2.38^a^	45.71±0.75^a^	31.73±0.67^a^	1.44±0.12^a^
CTX	52.15±1.61^c^	32.64±1.03^c^	28.53±0.36^c^	1.14±0.07^b^
LJP-L	53.37±1.28^c^	33.68±0.95^c^	29.34±0.62^bc^	1.15±0.09^b^
LJP-M	61.26±1.04^b^	38.17±0.88^b^	30.58±0.96^ab^	1.25±0.09^b^
LJP-H	74.83±1.31^a^	46.73±0.61^a^	30.49±1.12^ab^	1.53±0.13^a^

Results are mean ± standard deviation. Different lowercase letters in the same column within the same sample express the significant differences (*P* < 0.05).

## 4. Discussion

Immunosuppression is a manifestation of abnormal immune function in the body. Under the action of single or multiple pathogenic factors, the damages of body immune system will make it prone to infection or the formation of immune diseases. CTX is used as an effective chemotherapeutic drug in tumor treatment. In addition, CTX can treat a variety of immune diseases such as rheumatoid arthritis, lupus erythematosus and colitis [15–17]. However, as an immunosuppressive agent, CTX can also kill normal cells and decrease the body's immune function while treating diseases [18–20].

Polysaccharides are one of the basic building blocks of life. Many studies have found that plant polysaccharides can inhibit tumor growth, activate immune cells, and improve the body's immune function [21]. Moreover, plant polysaccharide is a natural nontoxic substance with various biological features. In a previous study, we isolated and characterized polysaccharides from *Lonicera Japonica*. The isolated polysaccharides were then injected into mice intraperitoneally with CTX (70 mg/kg) to establish an immunosuppressive mice model. Through a comparison between the model group and the normal control group, we found that CTX remarkable reduced the organ indices, the splenic lymphocyte proliferation, and the macrophage phagocytosis of mice. CTX also decreased splenic NK cytotoxicity and the serum IL-2, TNF-α, and IFN-γ concentration. Moreover, the percentage of CD4^+^ and CD8^+^ T cell subsets in CTX mice decreased significantly. The above results demonstrated a successful immunosuppressive mice model with CTX, which was consistent with previous reports [22–24].

The weight of immune organs is a reflection of body's innate immune function [25]. Thymus, a central immune organ, is the place for immune cells differentiation and maturity. Spleen is a peripheral immune organ, where mature immune cells colonize and respond to the immune response. Thus, changes in the thymus and spleen indices reflect the strength of body's innate immune function [26]. In this study, LJP remarkably increased the weight of spleen and thymus in the CTX-treated mice and enhanced the immune function of mice.

Lymphocytes are crucial cells in the adaptive immune response and lymphocyte proliferation experiment is often used to evaluate animal's immune response ability [27]. T lymphocytes and B lymphocytes are more sensitive to ConA and LPS, respectively. Therefore, we selected ConA and LPS to induce the proliferation of T and B lymphocytes, and examined the effects of LJP on the proliferation of splenic lymphocytes in immunosuppressed mice with different dosages. As a result, LJP-M and LJP-H markedly ameliorated the splenic lymphocyte proliferation of immunosuppressive mice induced by CTX, indicating that LJP is conducive to the activation of lymphocyte proliferation. These results are in accordance with previous studies [28–30].

Phagocytosis of macrophages is also a crucial indicator for the immune function of the body. Macrophages have a strong engulf particulate matter function *in-vivo* or *in-vitro* [31]. After mixing macrophages with particulate matter (such as CRBCs or *staphylococci*) for a certain period of time, the particulate matter can be phagocytosed by macrophages [32]. CRBCs, due to the presence of multiple protein complexes on their surface, can activate macrophages when injected into the abdominal cavity of mice. In our experiment, LJP enhanced the macrophages phagocytic rate and phagocytic index of the immunosuppression mice, especially in the LJP-H group, where the phagocytosis rate was increased by 30.38%.

NK cells are a major population of cytotoxic lymphocytes in the body. NK are not only related to anti-tumor, anti-viral infection and immune regulation, but also involved in the occurrence of hypersensitivity reactions and autoimmune diseases in some cases [33]. NK can directly recognize and kill the infected cells without the major histocompatibility complex (MHC) [34]. In this study, CTX reduced the NK cytotoxicity of immunosuppressed mice. The NK cytotoxicity of the LJP-M and LJP-H groups recovered gradually. This result demonstrated that LJP can enhance the immune function of immunosuppressed mice.

Cytokines mainly refer to a kind of small molecule polypeptides secreted by activated immune cells. As a signal transduction molecule between cells, cytokines can regulate immune response, participate in the development of immune cell differentiation, mediate inflammatory reaction, and stimulate hematopoietic function [35]. IL-2 is a cell growth factor that promotes cell proliferation and differentiation. IL-2 can induce the production of interferon, and involve in the process of inflammatory or autoimmune reactions [36]. TNF-α plays a very important role in the host defense mechanism and has a direct killing effect on tumor cells. TNF-α can also stimulate the expression of a series of immune mediators and inflammatory mediators [37]. The biological activities of IFN-γ are primarily to inhibit viral replication, activate macrophages, and induce the expression of MHC molecules [38]. As can be seen from our analyses, LJP had a good regulatory effect on CTX-induced immunodeficiency in mice. LJP raised the concentrations of IL-2, TNF-α and IFN-γ in mouse serum in a dose-dependent manner. The concentration of IL-2 and TNF-α in the LJP-H group returned to the levels in the control group. This result shows that LJP can adjust the body's immune imbalance and improve the symptoms of low immunity.

T lymphocytes are one of the most important immune cells in the body. T cell can be divided into different subsets based on different surface antigens and antibody marked on the T lymphocytes. The level of CD3^+^ T cell reflects the total level of T lymphocyte, while CD4^+^ and CD8^+^ are major functional subsets of T cell [39,40]. CD4^+^ T cell represents helper T cell that helps lymphocytes differentiate and produce antibodies. CD8^+^ T cell represents cytotoxic T cell that destroys infected cells. In healthy individuals, CD4^+^ and CD8^+^ T cells maintain a certain proportion (0.5–2) and participate cooperatively in the body's immune response. The change of CD4^+^/CD8^+^ ratio is considered as one of the important markers of human immune dysfunction [41]. Therefore, the determination of the CD4^+^, CD8^+^ T cell subsets, and the ratio of CD4^+^/CD8^+^ T can well reflect the body's immune function. In this experiment, LJP succeeded in raising the percentage of CD4^+^ and CD8^+^ T cell subsets and the ratio of CD4^+^/CD8^+^. The result demonstrated that LJP can directly enhance the cellular immune function in immunosuppressed mice.

In conclusion, our study demonstrated that LJP had the characteristic absorption of typical polysaccharides and consists of 6 types of monosaccharides. LJP can alleviate the negative influence on immune function in immunosuppression mice induced by CTX. These cytological, molecular and biochemical experimental results indicates that LJP can improve the immune functions and raise the antioxidant activities of immune organs in immunosuppression mice. Therefore, LJP could be used as an attractive immunomodulating agent. Our experimental results provides a basis data for further clinical application of LJP.
